# Para Além dos Fatores Biológicos: A Face Social das Doenças Cardiovasculares no Brasil

**DOI:** 10.36660/abc.20250550

**Published:** 2025-09-10

**Authors:** Lucas Borges Pereira, Maria Olívia Barboza Zanetti

**Affiliations:** 1 Universidade de São Paulo Faculdade de Ciências Farmacêuticas de Ribeirão Preto Ribeirão Preto SP Brasil Faculdade de Ciências Farmacêuticas de Ribeirão Preto – Universidade de São Paulo, Ribeirão Preto, SP – Brasil; 2 Faculdade de Ciências da Saúde de Barretos Dr. Paulo Prata Barretos SP Brasil Faculdade de Ciências da Saúde de Barretos Dr. Paulo Prata, Barretos, SP – Brasil

**Keywords:** Doenças Cardiovasculares, Determinantes Sociais da Saúde, Desigualdades de Saúde, Vulnerabilidade Social, Mortalidade

As doenças cardiovasculares (DCV), que incluem as doenças isquêmicas do coração (DIC) e as doenças cerebrovasculares (DCBV), representam a principal causa de morte globalmente e no Brasil.^1^ Embora avanços significativos tenham sido feitos na prevenção e tratamento, a mortalidade por DCV ainda exibe disparidades marcantes, frequentemente correlacionadas com indicadores socioeconômicos e de vulnerabilidade.^2 - 4^

Historicamente, a compreensão dos fatores de risco para DCV se concentrou em aspectos biológicos.^5^ No entanto, evidências apontam para o papel crucial dos Determinantes Sociais de Saúde (DSS), que englobam as condições sociais, econômicas, culturais, étnicas, educacionais e ambientais, na formulação de estratégias para o cuidado integral à saúde.^5 - 7^

O índice de vulnerabilidade social (IVS) foi desenvolvido pelo Instituto de Pesquisa Econômica Aplicada para quantificar a vulnerabilidade no Brasil. Ele é composto por 16 indicadores distribuídos em três dimensões principais: infraestrutura urbana (IVS-IU), capital humano (IVS-CH), e renda e trabalho (IVS-RT).^8^ O IVS fornece uma visão abrangente das fragilidades sociais, permitindo a identificação de áreas e populações mais vulneráveis. A compreensão de suas dimensões é fundamental para direcionar políticas públicas que visem à redução das iniquidades em saúde.

O estudo "Índice de Vulnerabilidade Social e Mortalidade por Doenças Isquêmicas do Coração e Doenças Cerebrovasculares no Brasil de 2000 a 2021" pretendeu analisar a evolução do IVS e suas dimensões, associado com as taxas de mortalidade por DIC e DCBV, no Brasil e nas unidades federativas (UF), no período de 2000 a 2021. De delineamento ecológico, utilizou dados do Sistema de Informações sobre Mortalidade e do Atlas de Vulnerabilidade Social.^9^

Este estudo mostrou que o IVS total, bem como as dimensões IVS-CH e IVS-RT apresentaram forte correlação com as taxas de mortalidade por DCBV e DIC. Em contraste, a dimensão IVS-IU apresentou uma correlação mais fraca com a mortalidade por DCBV.^9^ Embora a infraestrutura seja um componente importante da vulnerabilidade social, seu impacto direto sobre esses desfechos parece menos expressivo que o das dimensões capital humano, que incluem educação, saúde, renda e trabalho. Isso pode ser atribuída ao fato de que os principais determinantes das DCBV, como renda, escolaridade, condições laborais, estilo de vida e acesso a serviços de saúde, exercem influência mais direta e imediata sobre os comportamentos de risco, o acesso à saúde e a gestão de condições crônicas. Por outro lado, déficits estruturais como ausência de saneamento básico, água potável, iluminação pública, coleta de lixo e exposição à poluição impactam sobretudo doenças infecciosas, respiratórias, acidentes e violência, que, embora relevantes, não figuram entre os determinantes imediatos da mortalidade por DCBV.^10 , 11^ Ademais, avanços na infraestrutura básica em muitas regiões podem ter reduzido sua variabilidade ao longo do tempo.^12^

Utilizar outros indicadores sociais, como índice de Gini, que mede a desigualdade, ou os parâmetros que compõem as dimensões do IVS pode ajudar a compreender melhor por que capital humano e renda e trabalho se correlacionam mais fortemente com a mortalidade, visto que as três dimensões apresentam associação entre si. Nesse contexto, e diante das expressivas desigualdades regionais e sociais que caracterizam o Brasil, se torna imprescindível o uso estratégico e integrado das ferramentas de monitoramento epidemiológico disponíveis, como os dados do Sistema Único de Saúde (DATASUS), os levantamentos populacionais do IBGE (índice de Gini, censo, intercensos, projeções) e os índices produzidos por organismos como o PNUD (IDH, IDHM), para subsidiar políticas públicas de saúde cardiovascular mais equitativas e baseadas em evidências. Destaca-se também a necessidade de investir na capacitação de profissionais de saúde em ciência de dados e epidemiologia, ampliando sua habilidade de interpretar e aplicar esses indicadores na prática clínica e na gestão.

Os achados do estudo inferem que, apesar da melhora geral nos indicadores de IVS ao longo do período, as regiões Norte e Nordeste do Brasil permaneceram com os maiores níveis de vulnerabilidade em todas as dimensões. Além disso, populações negras e rurais apresentaram maior vulnerabilidade, e a população feminina mostrou maior vulnerabilidade na dimensão IVS-RT.^9^ À vista disso, embora as políticas públicas de promoção à saúde cardiovascular devam contemplar toda a população, devem incluir estratégias de triagem e cuidado mais sensíveis às desigualdades sociais, com atenção especial a grupos historicamente mais vulneráveis, como populações negras, rurais, das regiões Norte e Nordeste, e mulheres em situação de maior vulnerabilidade socioeconômica, frequentemente agravada por desigualdade de gênero, sobrecarga de cuidados e menor acesso a recursos econômicos e de saúde.^13^

O artigo reforça que boas condições de vida (socioeconômicas e educacionais) estão intimamente ligadas a melhores desfechos cardiovasculares. Para mitigar o fardo das DCVs no Brasil, é importante que as políticas públicas considerem os DSS. Isso inclui investimentos em educação, geração de emprego e renda, e a garantia de acesso equitativo a serviços de saúde de qualidade. A identificação e o enfrentamento das desigualdades sociais são passos fundamentais para reduzir as disparidades na saúde cardiovascular e promover um bem-estar mais abrangente para toda a população brasileira.
