# Supplementary material for: Para Além dos Fatores Biológicos: A Face Social das Doenças Cardiovasculares no Brasil
Source: Arq Bras Cardiol. 2025 Sep 10;122(8):e20250550. [Article in Portuguese] doi: 10.36660/abc.20250550 (PMC12671609; doi:10.36660/abc.20250550)
Supplement: Translation

# Translation: Beyond Biological Factors: The Social Face of Cardiovascular Disease in Brazil

**DOI:** 10.36660/abc.20250550i

**Authors:** Lucas Borges Pereira, Maria Olívia Barboza Zanetti

**Affiliations:** 1 Ribeirão Preto SP Brazil Faculdade de Ciências Farmacêuticas de Ribeirão Preto – Universidade de São Paulo, Ribeirão Preto, SP – Brazil; 2 Barretos SP Brazil Faculdade de Ciências da Saúde de Barretos Dr. Paulo Prata, Barretos, SP – Brazil

**Keywords:** Cardiovascular Diseases, Social Determinants of Health, Health Inequities, Social Vulnerability, Mortality

Cardiovascular diseases (CVD), which include ischemic heart disease (IHD) and cerebrovascular diseases (CVD), represent the leading cause of death globally and in Brazil.^1^Although significant advances have been made in prevention and treatment, CVD mortality still exhibits striking disparities, often correlated with socioeconomic and vulnerability indicators.^2 - 4^

Historically, understanding CVD risk factors has focused on biological aspects.^5^However, evidence points to the crucial role of Social Determinants of Health (SDH), which encompass social, economic, cultural, ethnic, educational, and environmental conditions, in formulating strategies for comprehensive health care.^5 - 7^

The Institute of Applied Economic Research developed the Social Vulnerability Index IVS ( *Índice de Vulnerabilidade Social* ) to quantify vulnerability in Brazil. It consists of 16 indicators distributed across three main dimensions: urban infrastructure (IVS-IU), human capital (IVS-CH), and income and labor (IVS-RT).^8^The IVS provides a comprehensive view of social fragilities, allowing the identification of more vulnerable areas and populations. Understanding its dimensions is essential for directing public policies aimed at reducing health inequities.

The study " Social Vulnerability Index and Mortality from Ischemic Heart Diseases and Cerebrovascular Diseases in Brazil from 2000 to 2021" aimed to analyze the evolution of the IVS and its dimensions, associated with mortality rates from IHD and CBVD, in Brazil and the federative units (UF), in the period from 2000 to 2021. With an ecological design, it used data from the Mortality Information System and the Social Vulnerability Atlas.^9^

This study showed that the total IVS, as well as the IVS-CH and IVS-RT dimensions, strongly correlated with CBVD and IHD mortality rates. In contrast, the IVS-IU dimension had a weaker correlation with CBVD mortality.^9^Although infrastructure is an important component of social vulnerability, its direct impact on these outcomes appears less significant than that of the human capital dimensions, which include education, health, income, and work. This may be attributed to the fact that the main determinants of CBVD, such as income, education, working conditions, lifestyle, and access to health services, exert a more direct and immediate influence on risk behaviors, access to health care, and the management of chronic conditions. On the other hand, structural deficits such as the lack of basic sanitation, drinking water, public lighting, garbage collection, and exposure to pollution have a particular impact on infectious and respiratory diseases, accidents, and violence, which, although relevant, are not among the immediate determinants of mortality from CBVD.^10 , 11^Furthermore, advances in basic infrastructure in many regions may have reduced its variability over time.^12^

Using other social indicators, such as the Gini index, which measures inequality, or the parameters that comprise the IVS dimensions, can help better understand why human capital, income, and labor correlate more strongly with mortality, given that these three dimensions are interrelated. In this context, and given the significant regional and social inequalities that characterize Brazil, the strategic and integrated use of available epidemiological monitoring tools, such as data from the Unified Health System (DATASUS), population surveys from the IBGE (Gini index, census, intercensuses, projections), and indices produced by organizations such as the UNDP (HDI, HDI-M), is essential to support more equitable and evidence-based public cardiovascular health policies. It is also important to invest in training healthcare professionals in data science and epidemiology, expanding their ability to interpret and apply these indicators in clinical practice and management.

The study's findings infer that, despite the overall improvement in IVS indicators over the period, the North and Northeast regions of Brazil remained with the highest levels of vulnerability across all dimensions. Furthermore, Black and rural populations were more vulnerable, and the female population showed greater vulnerability in the IVS-RT dimension.^9^Therefore, although public policies to promote cardiovascular health should encompass the entire population, they should include screening and care strategies that are more sensitive to social inequalities, with special attention to historically more vulnerable groups, such as Black, rural populations in the North and Northeast regions, and women in situations of greater socioeconomic vulnerability, often aggravated by gender inequality, care burden, and reduced access to economic and health resources.^13^

The article reinforces that good living conditions (socioeconomic and educational) are closely linked to better cardiovascular outcomes. To mitigate the burden of CVDs in Brazil, public policies must consider the SDH. This includes investments in education, job and income generation, and ensuring equitable access to quality health services. Identifying and addressing social inequalities are fundamental steps toward reducing disparities in cardiovascular health and promoting broader well-being for the entire Brazilian population.
